# The vascular endothelial growth factor (VEGF) receptor-2 is a major regulator of VEGF-mediated salvage effect in murine acute hepatic failure

**DOI:** 10.1186/2040-2384-2-16

**Published:** 2010-08-24

**Authors:** Tadashi Namisaki, Hitoshi Yoshiji, Ryuichi Noguchi, Yasuhide Ikenaka, Mitsuteru Kitade, Kosuke Kaji, Yusaku Shirai, Yosuke Aihara, Junichi Yoshii, Koji Yanase, Tatsuhiro Tsujimoto, Hideto Kawaratani, Hiroshi Fukui

**Affiliations:** 1 Third Department of Internal Medicine, Nara Medical University, Shijo-cho 840, Kashihara, Nara 634-8522, Japan

## Abstract

Although administration of the vascular endothelial growth factor (VEGF), a potent angiogenic factor, could improve the overall survival of destroyed sinusoidal endothelial cells (SEC) in chemically induced murine acute hepatic failure (AHF), the mechanistic roles of the VEGF receptors have not been elucidated yet. The respective roles of VEGF receptors; namely, Flt-1 (VEGFR-1: R1) and KDR/Flk-1 (VEGFR-2: R2), in the D-galactosamine (Gal-N) and lipopolysaccharide (LPS)-induced AHF were elucidated with specific neutralizing monoclonal antibody against R1 and R2 (R1-mAb and R2-mAb, respectively). The serum ALT elevation, with a peak at 24 h after Gal-N+LPS intoxication, was markedly augmented by means of the R1-mAb and R2-mAb. The aggregative effect of R2-mAb was more potent than that of R1-mAb, and the survival rate was 70% in the R2-mAb-treated group and 100% in the other groups. The results of SEC destruction were almost parallel to those of the ALT changes. Our in-vitro study showed that R1-mAb and R2-mAb significantly worsened the Gal-N+LPS-induced cytotoxicity and apoptosis of SEC mediated by caspase-3, which were almost of similar magnitude to those in the in-vivo study. In conclusion, these results indicated that R2 is a major regulator of the salvage effect of VEGF on the maintenance of SEC architecture and the anti-apoptotic effects against chemically-induced murine AHF.

## Background

Despite the recent advances in liver support systems, acute hepatic failure (AHF) still has a high mortality rate [1]. Among several types of non-parenchymal cells, the sinusoidal endothelial cells (SEC) are considered the most important in the recovery from AHF [2]. The initial wave of hepatocyte proliferation is followed by SEC proliferation and penetration of avascular hepatocellular islands leading to formation of new sinusoids [3]. Several studies have proven that neovascularization requires these processes during the recovery from AHF [4].

Angiogenesis is the development of new vasculature from the pre-existing blood vessels and/or the circulating EC stem cells [5,6]. Emerging evidences have shown that angiogenesis plays a pivotal role in many physiological and pathological processes, such as tumor growth, arthritis, psoriasis, and diabetic retinopathy [5,7]. Angiogenesis is regulated by the net balance between pro-angiogenic factors and angiogenic inhibitors. To date, many positive and negative angiogenic-modulating factors have been identified. Among these, the vascular endothelial growth factor (VEGF) is the most potent factor in the angiogenesis process [8]. Emerging evidences have shown that VEGF plays a pivotal role in many processes of physiological and pathological angiogenesis [9]. VEGF is not only an angiogenic factor but also known as a survival factor for EC [10]. Regarding liver regeneration, it has been shown that the VEGF expression increased markedly during liver regeneration induced either by partial hepatectomy (PH) or drug intoxication [11]. Furthermore, exogenous VEGF administration after PH promoted the proliferative activity in the liver [12]. Conversely, it has shown that neutralization of VEGF significantly inhibited the proliferative activity in the liver during regeneration after PH [13]. In addition to the vitality of regeneration, we previously reported that the VEGF-mediated maintenance of the SEC architecture through anti-apoptotic effects in AHF is important. VEGF treatment significantly reduced the mortality rate of AHF in the rat through maintenance of the SEC architecture and anti-apoptotic effect on SEC [14].

The biological effects of VEGF are mediated by two receptor tyrosine kinases; namely, Flt-1 (VEGFR-1: R1) and KDR/Flk-1 (VEGFR-2: R2), which differ considerably in the signaling properties [15]. Both VEGFRs are expressed almost exclusively on the surface of EC. R1 activation resulted in paracrine release of the hepatocyte growth factor (HGF), interleukin-6 (IL-6), and other hepatotrophic molecules from SEC, and the hepatocytes were stimulated to proliferate when cultured with SEC [16]. R2 activation led to an increase in proliferation of EC after hepatic injury, that in turn, led to EC regeneration. It has already been shown that neutralization of VEGF with anti-VEGF antibody significantly inhibited the proliferative activity in liver regeneration after PH [13]. And that the specific neutralizing monoclonal antibody against R2 (R2-mAb) would impair liver regeneration in mice [17]. Using R-2mAb, we previously found that R2 was a major regulator of VEGF-mediated tumor development and angiogenesis in several animal models [18,19]. However, the respective roles of the VEGF receptors in AHF have not been elucidated yet.

In the current study, we elucidated the respective roles of R1 and R2 in the Gal-N+LPS-induced AHF using specific neutralizing monoclonal antibody for R1 and R2, especially in conjunction with maintenance of the SEC structure.

## Methods

### Reagents and animal treatment

Ten-week-old male Balb/c mice weighing (18-20 g) obtained from Japan SLC Inc (Hamamatsu, Shizuoka, Japan) were used. They were housed in stainless steel, mesh cages under controlled conditions of temperature (23 ± 3°C) and relative humidity (50 ± 20%), with 10-15 air changes per hour and light illumination for 12 hours (h) a day. The animals were allowed access to food and tap water *ad libitum *throughout the acclimatization and experimental periods. D-galactosamine hydrochloride (Gal-N) was purchased from Nacalai Tesque (Kyoto, Japan) and *Escherichia coli endotoxin *(LPS, serotype 055:B5) was purchased from Sigma Chemical (St. Louis, MO, USA). The anti-R1 and anti-R2 specific neutralizing monoclonal antibodies (R1-mAb and R2-mAb, respectively) were generated as described previously [20]. Briefly, hybridoma cells were grown via continuous feed fermentation in a serum-free medium. mAbs were purified from conditioned media by a multistep chromatography process and assessed for purity in SDS-PAGE, and the immunoreactivity with soluble R-1 and R-2 receptors was checked by ELISA. The negative control polyclonal rat IgG was purchased from Jackson ImmunoResearch Laboratories (West Grove, PA, USA). mAbs and control rat IgG were tested for endotoxin using the Pyrogent Plus *Limulus *amebocyte lysate kit (BioWhittaker, Walkersville, MD, USA). All antibody preparations used in animal studies contained ≤1.25 endotoxin units/ml of endotoxin. It has been shown that R2-mAb exerted a VEGFR2 inhibitory effect in a dose-dependent manner, and that the maximal effect was achieved at a dose of 800 μg/mouse administered twice a week [21,22]. We therefore employed this dose in the current study. The mice were randomly divided into four groups. Group 1 (G1) consisted of the phosphate buffer saline (PBS) and the control immunoglobulin-G (IgG)-treated mice and served as a control group. The other mice were injected with Gal-N (375 mg/kg) and LPS (50 μg/kg) intraperitoneally. Since the survival rate by combination treatment with 500 mg/kg of Gal-N and LPS (50 μg/kg) was 27% [14,23], we decreased the dose of Gal-N in the current study to induce a moderate liver injury. Mice in group 2 (G2) received the control IgG at 0 h. Animals in group 3 (G3) and group 4 (G4) received equal amounts of R1mAb or R2mAb intraperitoneally at 0 h, respectively. Liver injury was evaluated by measuring the serum alanine aminotransferase (ALT), which reaches a peak at 24 h [14,23]. Fifteen mice from each experimental group were used for monitoring the survival rate chronologically. Ten mice from each group were killed at 24 h under anesthesia. Another experiment was performed to investigate the survival rate (n = 10, each group). Blood samples were withdrawn via the abdominal aorta. All animal procedures were performed according to approved protocols and in accordance with the standard recommendations for the proper care and use of laboratory animals.

### Immunohistochemistry

To elucidate the effects of mAbs on the maintenance of SEC structure at 24 h after administration of Gal-N+LPS, an immunohistochemical analysis of ICAM-1 was performed as described previously [24]. The livers from five mice in each group were carefully excised at 24 h after intoxication. Then, 5-μm thick slices from the major liver lobes were fixed in ice-cold acetone and embedded in paraffin. Serial sections were prepared from each fixed liver. The first was routinely stained with hematoxylin and eosin for histological examination. The other sections were used for immunohistochemical analysis. The immunopositive SEC were evaluated with Adobe Photoshop and NIH image software as previously described [25].

### In-vitro cytotoxicity and apoptosis of EC

The *in-vitro *cytotoxicity of EC treated with Gal-N (20 mM) and LPS (100 mg/ml) was determined by MTT assay, which reflected the mitochondrial activity as described elsewhere (n = 6 per group) [26]. The effects of mAbs treatment on the EC apoptosis were examined by the Cell Death Detection ELISA kit (Roche, Tokyo, Japan) according to the manufacturer's instructions as described previously [27]. We also examined the activity of caspase-3 which is apoptosis regulatory protein by Caspase-3/CPP32 Colorimetric Assay Kit (BioVision Research Products, CA, USA) according to the manufacturer's instructions.

### Statistical analysis

The data are presented as means ± SD. The statistical significance of differences was evaluated by one-way analysis of variance followed by Barlett's test for comparisons among four or eight means. A p value of <0.05 was considered statistically significant.

## Results

### Effects of VEGFR-mAbs on the serum ALT and survival rate

**Figure 1:**
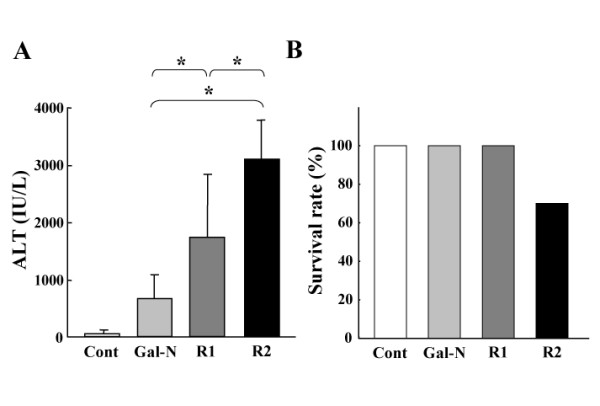
**The effects of R1-mAb and R2-mAb on the serum ALT level and survival rate of rats with Gal-N+LPS-induced acute hepatic failure**. A: The effects of R1-mAb and R2-mAb on the serum ALT level following Gal-N+LPS intoxication. Injection of Gal-N+LPS resulted in a moderate increase of the serum ALT level at 24 h. Both R1-mAb and R2-mAb significantly augmented the elevation of ALT, and the elevation magnitude with R2-mAb was significantly higher than with R1-mAb. B: The survival rate 24 h after intoxication in relation to the peak of ALT. The survival rate in the R2mAb-treated animals was 70%, whereas no animals died in the other groups. No mice died after 24 h in all groups. Cont: Control IgG-treated mice (G1). Mice in group 2 (G2: Gal-N) simultaneously received Gal-N (375 mg/kg), LPS (50 μg/kg), and the control IgG intraperitoneally at 0 h. Instead of the control IgG, animals in group 3 (G3: R1) and group 4 (G4: R2) received the equal amounts of R1mAb or R2mAb intraperitoneally at 0 h, respectively. Each points in Fig. 1A represents the mean ± SD. *: Statistically significant differences between the experimental groups (p < 0.05).

The effects of the mAbs treatment on the Gal-N+LPS-induced ALT elevation and survival rate in AHF were first examined. As shown in Fig. 1A, the serum ALT levels in the mAbs-treated groups were markedly higher than in the Gal-N-treated group at 24 h after Gal-N+LPS intoxication. The ALT level with R2-mAb was significantly higher than with R1-mAb. We also examined the survival rate at 24 h after intoxication in relation to the peak of ALT as described previously [14,23]. In the control (G1), the Gal-N-treated (G2), and R1-mAb-treated (G3) groups, all animals survived. On the other hand, the survival rate in the R2mAb-treated animals (G4) was 70% (Fig. 1B). No mice died after 24 h in all groups.

### Effects of VEGFR-mAbs on the maintenance of SEC architecture

**Figure 2:**
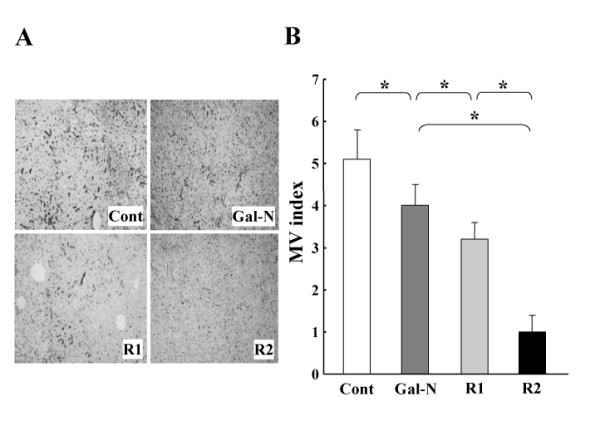
**The effects of R1-mAb and R2-mAb on the maintenance of EC structure of rats with Gal-N+LPS-induced acute hepatic failure**. A: Representative features of the effects of R1-mAb and R2-mAb on maintenance of the SEC structure after intoxication by combination of Gal-N+LPS. The R1-mAb and R2-mAb treatment significantly augmented the destruction of the SEC architecture in the liver after Gal-N+LPS intoxication. The SEC architecture was evaluated by the ICAM-1-immunopositive vessels. Original magnification: × 40. B: Semi-quantitative analysis of the SEC architecture in the liver following the Gal-N+LPS intoxication. Individual treatment with R1-mAb and R-2mAb significantly augmented the destruction of the SEC architecture in the liver both at 24 h after intoxication. The destruction magnitude of R2-mAb was significantly higher than that of R-1 mAb. Cont: Control IgG-treated mice (G1). Mice in group 2 (G2: Gal-N) simultaneously received Gal-N (375 mg/kg), LPS (50 μg/kg), and the control IgG intraperitoneally at 0 h. Instead of the control IgG, animals in group 3 (G3: R1) and group 4 (G4: R2) received equal amounts of R1mAb or R2mAb intraperitoneally at 0 h, respectively. MV: microvessel. The data represent the mean ± SD. *: Statistically significant differences between the experimental groups (p < 0.05).

We next examined the effects of R1-mAb and R2-mAb on the maintenance of SEC architecture after intoxication by combination of Gal-N+LPS. We previously observed that the initial SEC construction in the untreated group was almost obliterated at 24 h after intoxication [14,23]. In this study, we elucidated the effects of mAbs at 24 h. The R1-mAb and R2-mAb treatment significantly augmented the destruction of the SEC architecture in the liver after Gal-N+LPS intoxication (Fig. 2A). Similar to the ALT level, the destruction magnitude with R2-mAb treatment was significantly higher than that with R-1 mAb. Semiquantitative analysis of the IACM-1-positive vessels confirmed these results (Fig. 2B). We examined the mRNA expression of ICAM-1, and observed that the mRNA expression of ICAM-1 was almost similar to that of the immunohistochemical analysis. Moreover, we also investigated tyrosine-phosphorylated VEGFR-1 and VEGFR-2 in the liver after i.p. injection of R-1mAb and R-2mAb. We confirmed that the R-1mAb and R-2mAb significantly inhibited tyrosine-phosphorylation of the respective receptors (data not shown).

### In-vitro studies

**Figure 3:**
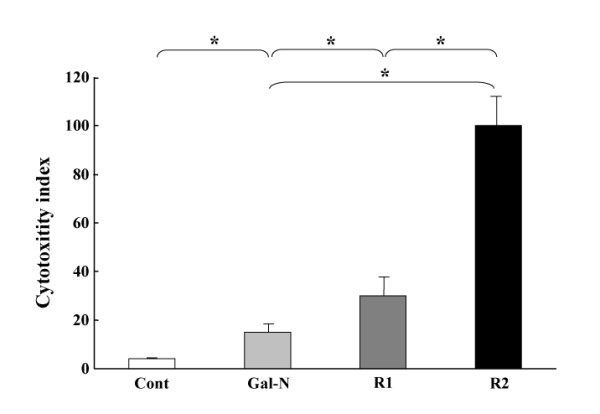
**Effects of R1-mAb and R2-mAb on the in-vitro Gal-N+LPS-induced cytotoxicity of EC**. The cell cytotoxicity was measured by the MTT assay as described in the Materials and Methods. The R1-mAb and R2-mAb treatment significantly augmented the cytotoxicity of EC. Moreover, the effect of R2-mAb was much more potent than that of R1-mAb. Cont: Control IgG-treated group. R1 and R2: R1-mAb- and R2-mAb-treated group, respectively. The data represent the mean ± SD. *: Statistically significant differences between the experimental groups (p < 0.05).

**Figure 4:**
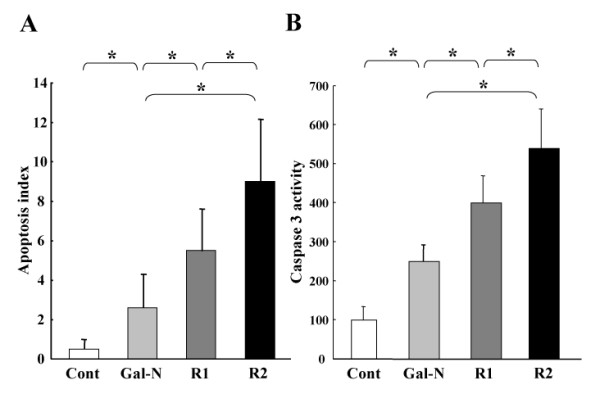
**Effects of R1-mAb and R2-mAb on the in-vitro apoptosis and caspase-3 activity of EC**. A: The R1-mAb and R2-mAb significantly increased the apoptotic index induced by Gal-N+LPS. The effect of R2-mAb on the apoptotic index was also more potent than R1-mAb. The effects of mAbs on the caspase-3 were almost in parallel with the augmentation of the apoptotic index (B). Cont: Control IgG-treated group. R1and R2: R1-mAb- and R2-mAb-treated group, respectively. The data represents the mean ± SD (n = 6). *: Statistically significant differences between the experimental groups (p < 0.05).

A set of *in-vitro *experiment was performed to assess the effects of mAbs on cytotoxicity and apoptosis induced by Gal-N+LPS. As shown in Fig. 3, the R1-mAb and R2-mAb treatment significantly augmented the cytotoxicity of EC. Moreover, the effect of R2-mAb was much more potent than that of R1-mAb. We also elucidated the effects on the apoptosis. Similar to the results of cytotoxicity, R1-mAb and R2-mAb treatment significantly increased the apoptotic index induced by Gal-N+LPS. The effect of R2-mAb on the apoptotic index was also more potent than that of R1-mAb (Fig. 4A). To examine the possible signaling cascade of apoptosis, we measured the caspase-3 activity, which is known as one of the apoptosis-regulatory key proteins, by colorimetric assay. The effects of mAbs on the caspase-3 were almost in parallel with the augmentation of the apoptotic index, indicating that the apoptosis signaling induced by R1-mAb and R2-mAb was mainly mediated by the caspase-3 cascade (Fig. 4B).

## Discussion

Angiogenesis is a complex and critical process in the development, growth, wound healing, and regeneration. Recent studies have revealed that these processes commonly occur together in many disease states, where neovascularization is believed to initiate the pathological cascade [28]. Among the identified angiogenic factors to date, VEGF is one of the most potent and central factors in many physiological and pathological processes [15,29,30]. VEGF is now recognized as a survival factor for EC as well as a major regulator of angiogenesis. We previously reported that recombinant VEGF administration markedly reduced the mortality rate of AHF in the rat through maintenance of the SEC architecture, by attenuating cytotoxicity and apoptosis of EC [23].

The biological activities of VEGF are mediated mainly via two type III tyrosine kinase receptors; namely, R1 and R2, which serve different roles in the angiogenesis and signal transduction pathways [15,29,31,32]. Although R1 shows a high affinity to VEGF, at least 10-fold higher than R2, R2 is a major positive mitogenic signal transducer through its strong kinase activity as compared with R1 [9]. Overexpression of R2 in the porcine EC led to actin reorganization, chemotaxis, and mitogenesis in response to VEGF, though R1 expression in the same cells had a minimal effect *in vitro *[31]. However, recent studies have revealed that R1 was also involved in the pathological angiogenesis, such as tumor growth [22,33-36]. In the present study, we found that either inhibition of R1 or R2 significantly augmented the SEC destruction, and that treatment with R2-mAb was more potent than that with R1-mAb. Furthermore, only R-2mAb treatment was lethal but not R1-mAb in the Gal-N+LPS-induced murine AHF. These results indicated that VEGF-VEGFR interaction was a major regulator in the maintenance of the SEC architecture in Gal-N+LPS-induced AHF.

It would be an important issue to elucidate the localization of R1-mAb and R2-mAb during this process. It is, however, difficult to make marking on these mAbs for immunohistochemical analysis at this time. We tried a couple of times to localize R1 and R2 by immunohistochemical double-staining with ICAM-1, but we could not get good results. The background was very intense, and the interpretation was very difficult (data not shown). We previously utilized SE-1 as a marker of SEC, as SE-1 is reportedly a specific marker of the liver SEC [37]. However, we could not apply SE-1 in the current study, because this antibody was only specific for the rat but not the mouse. We could not obtain any specific R1-mAb and R2-mAb for the rat at this time. When the mAbs against the rat R1 and R2 become available, further studies would be required with these mAbs and SE-1 to elucidate the localization of respective receptors in AHF.

Regarding the salvage effect of VEGF against AHF, inhibition of apoptosis in EC was also a prerequisite [23]. There is now a consensus that R2 is the major mediator of the anti-apoptotic as well as the mitogenic effects of VEGF. R2 and PI3-kinase/Akt signal transduction pathway as a crucial element in the processes leading to endothelial cell survival induced by VEGF [38]. Recent studies on R1 have also detected a pro-survival signal in ECs, possibly mediated by induction of the anti-apoptotic gene that survives under some circumstances [9]. Furthermore, it has been reported that the transplanted EPC secreted growth factor in a paracrine manner and inhibited cell apoptosis through R1 and R2. Similar to the *in-vivo *studies, we found that inhibition of either R1-mAb or R2-mAb significantly augmented the apoptosis. In addition, treatment with R2-mAb was more potent than that with R1-mAb, indicating that VEGF-R2 interaction was a major regulator of the anti-apoptotic effect as well as the maintenance of the SEC architecture. Concerning the apoptotic signaling, it has been reported that there is differential involvement of Akt and Erk1/2 in apoptosis and proliferation signal transduction between R1 and R2 [39]. Although we found that the caspase-3 activity was almost similar to that of the total apoptotic index, further studies are required utilizing selective signal pathway inhibitors to determine the downstream nuclear protein.

Since the impaired liver regeneration is one of the most critical issues in the prognosis, VEGF is considered to play an important role in hepatic regeneration via maintenance of the SEC architecture, attenuating cytotoxicity and anti-apoptosis for EC. It has been reported that the cross-talk between SEC and hepatocytes played a critical role in liver regeneration following toxic injury [3]. The increased expressions of VEGF and VEGFR correlated with the rate of SEC proliferation after PH [40]. R2 is a major positive mitogenic signal transducer through its strong kinase activity as compared with R1 [9]. R2 activation not only mediated SEC proliferation but also resulted in induction of a subset of hepatotrophic genes [41]. It has been shown that R1 is also an important mediator in bone marrow-derived EC progenitor cell recruitment [22], and this type of cells reportedly contributes to liver regeneration [13]. Moreover, it has been reported that the VEGF-mediated paracrine effect on SEC through R2 was involved in maintenance of the SEC phenotype [42]. These co-ordinate effects by VEGF and VEGFR interaction should contribute to decrease the overall survival of the R2-mAb-treated group with AHF in the current study.

In conclusion, we observed that VEGF-VEGFRs interaction was a prerequisite against chemically-induced murine AHF, and that R2 was a major regulator of maintenance of the SEC architecture by attenuating the cytotoxicity and apoptosis of EC more than R1. In addition to VEGF administration, stimulation of VEGFRs, especially R2, such as during gene delivery, would be an alternative new therapeutic strategy for AHF in the future.

## Competing interests

The authors declare that they have no competing interests.

## Authors' contributions

HY conceived of the study, carried out the main body of the project and prepared the manuscript. TN, RN, YI, MK, KK, YS, YA, JY, KY, HK, TK, and HF participated in the most part of the studies such as animal handling and sample analysis. All authors read and approved the final manuscript.
